# Diseases of Children

**Published:** 1886-11-06

**Authors:** 


### Till the Doctor Comes.

[Inquiries and Communications for this column should be addressed, "Physician," care of the Editor of The Hospital.]

### III.—DISEASES OF CHILDREN.

Constipation. If the child is very young, a teaspoonful of glycerine every morning will often succeed in overcoming it, or rubbing the bowels night and morning with sweet oil. Sometimes very small doses of belladonna, given every night as a parvule, will in time produce a proper action. In older children fruit may be tried, or the diet may be varied.

Diarrhœa and Vomiting. Generally due to faulty feeding. If it shows signs of continuing, do not neglect it, but seek medical advice at once. On its first appearance give only milk, or milk and lime water in proportions, according to the age, with a few drops of brandy; give more meals, and less quantities at each meal. If the child has habitually much flatulence, a few drops of brandy will do good.

Disease of the Lungs. In bronchitis it is very necessary to keep up a warm moist atmosphere whilst the attack lasts, and the child should remain continuously in one room at a certain temperature, with a bronchitis kettle at work, or some hot water poured occasionally into a bucket standing near the bed, so that the steam may escape into the room. It is of no use to simply keep a kettle boiling on the fire, as the steam goes then straight up the chimney; a tube must be put to the spout of the kettle, so as to convey the steam into the room. Inflammation of the lungs in children is generally a further stage of bronchitis.

Croup. The most common form of croup is due to inflammation of the upper part of the windpipe, and is dangerous from the small size of this tube in children, so that the swelling caused by the inflammation may quite block it. It is the same cause that renders bronchitis in children so much more dangerous than in grown-up people. In this disease the precautions given for bronchitis must be emphasized. A tent must be made by tying four poles, each about six feet long—one to each corner of the bed—and spreading sheets or blankets over them, so as to leave only one opening for access to the child; the tube from the kettle must be led into this tent, and the air inside is thus kept warm and moist. Croup is known by the hoarse voice or total loss of voice, the peculiar noise made in breathing, and the harsh paroxysms of cough; almost every parent is familar with the sound. Always give a hot bath immediately, which often affords effectual relief. Should the breathing get worse, however, lay some sponges dipped in water as hot as they can be borne over the throat just above the breast bone. It is a common custom to give ipecacuanha wine till the child is sick, but in every case at all severe, get a doctor at once; soothing medicine and patience do just as much good as emetics. The child usually gets better the next day; but do not be deceived, continue all precautions, for the breathing is almost sure to get worse again at night for some few nights.

**A Warning.** It must be mentioned that there is another form of croup, happily very rare, which is exceedingly dangerous, and almost always fatal. It is, however, impracticable to describe it, and it is only mentioned here to show how necessary it is to procure the early attendance of a doctor. Till he arrives the treatment above given for croup is all that can be done.

**Scald of Windpipe.** Not very infrequent. It occurs thus: A young child tries to drink water out of the spout of a kettle, or some vessel containing boiling water. The moment the water gets into the mouth, the pain causes the child to shriek loudly, during which he takes a deep breath, and the water is drawn into the windpipe. It corresponds in symptoms and treatment almost exactly with the most severe forms of croup.

**Convulsions.** Only too common, and need no description. Send for a doctor, and till he arrives make a minute examination of the child, to see if you can detect any source of irritation that may account for the fit. If you can remove it, the convulsions will probably soon cease; a pin may be pricking the child, it may be due to the teeth, etc. Undress the child and put it in a hot bath (100° F.), and put some cold rags to its head. Keep it in the bath twenty minutes, and whether the convulsions cease or not, wrap it then in a blanket, and put it into bed; keep it perfectly quiet in a darkened room, and avoid all noise, talking, etc.; continue the cold rags to the head. A convulsion is often the prelude in children to an attack of some severe disease, as scarlatina, pneumonia, etc.

*(To be continued.)*

